# Instrumentation to study myofibril mechanics from static to artificial simulations of cardiac cycle

**DOI:** 10.1016/j.mex.2016.02.006

**Published:** 2016-03-02

**Authors:** Petr G. Vikhorev, Michael A. Ferenczi, Steven B. Marston

**Affiliations:** a National Heart and Lung Institute, Imperial College London, London, United Kingdom; b Lee Kong Chian School of Medicine, Nanyang Technological University, Singapore, Singapore

**Keywords:** Muscle contraction, Myofibril contractility, Myofibril mechanics, Cardiac cycle simulation, Biomedical instrumentation, LabVIEW software

## Abstract

Many causes of heart muscle diseases and skeletal muscle diseases are inherited and caused by mutations in genes of sarcomere proteins which play either a structural or contractile role in the muscle cell. Tissue samples from human hearts with mutations can be obtained but often samples are only a few milligrams and it is necessary to freeze them for storage and transportation. Myofibrils are the fundamental contractile components of the muscle cell and retain all structural elements and contractile proteins performing in contractile event; moreover viable myofibrils can be obtained from frozen tissue.

• We are describing a versatile technique for measuring the contractility and its Ca^2+^ regulation in single myofibrils. The control of myofibril length, incubation medium and data acquisition is carried out using a digital acquisition board via computer software. Using computer control it is possible not only to measure contractile and mechanical parameters but also simulate complex protocols such as a cardiac cycle to vary length and medium independently.
• This single myofibril force assay is well suited for physiological measurements. The system can be adapted to measure tension amplitude, rates of contraction and relaxation, Ca^2+^ dependence of these parameters in dose-response measurements, length-dependent activation, stretch response, myofibril elasticity and response to simulated cardiac cycle length changes. Our approach provides an all-round quantitative way to measure myofibrils performance and to observe the effect of mutations or posttranslational modifications. The technique has been demonstrated by the study of contraction in heart with hypertrophic or dilated cardiomyopathy mutations in sarcomere proteins.

## Graphical abstract

**Figure:**
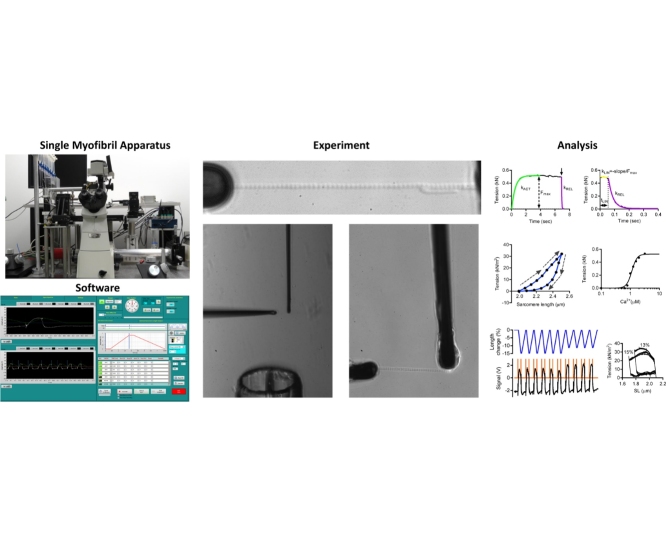

## Method details

Many experimental systems have been used for the study of muscle contraction. Single molecule or single filament type assays allow unlimited control over incubating medium and protein composition but do not have the structural organization of a muscle. Chemically skinned muscle cells or fibers allow for the measurement of contractility but response to changes in the medium is limited by diffusion and results may be confused by non-sarcomeric elastic elements. The optimum preparation for studying contractility is the myofibril – the fundamental unit of striated muscle. Myofibrils are ∼1 μm in diameter and up to several 100 μm long. Myofibrils are built up from repeating contractile subunits called sarcomeres, composed of actin and myosin filaments arranged in parallel. Powered by energy of ATP, myosin heads push actin filaments alongside causing actin and myosin filaments to slide past each other. Such process results in shortening of sarcomeres, myofibrils and the whole muscle cell. The troponin complex together with tropomyosin, which sterically blocks interaction of myosin with actin at low Ca^2+^ concentration, controls this process [1]. Not much is yet known about the modulation of this process by other sarcomeric proteins, such as myosin binding protein C, titin, nebulin and obscurin. But there is no doubt that this is of physiological importance [2], [3], [4], [5], [6]. It was found that the most causes of heart diseases and muscular dystrophies are inherited and caused by mutations in genes of sarcomere proteins [7], [8]. What is more, changes in isoform protein expression, phosphorylation [9] and oxidation [10] also affect myofibril contractility in several ways. The muscle diseases not only diminish the quality of life but also are one of the highest causes of death. Comprehensive experimental studies of the mechanisms leading to the diseases are important as for the choice of treatment as well as for the discovery of new ones [11], [12].

Myofibrils can be isolated in sufficient quantities from only a few milligrams of fresh or frozen tissue samples or cells meaning that the sources are potentially unlimited. The preparation preserves native organization of sarcomeric and structural proteins. The use of myofibrils in contractile experiments has many practical advantages compared to the use of permeabilised muscle fibers. Because of myofibrils smaller diameter there are no diffusional limitations (<1 ms) and rapid solution changes are easy so that the quick processes of contraction and relaxation can be studied. Dynamics of individual sarcomeres can be followed. However, the small size of myofibrils requires special apparatus.

The early apparatus design to measure force in single myofibrils can be dated back to the papers of Iwazumi [13] and Cecchi et al. [14], [15] that also provide the most detailed description of the system. Another successful apparatus setup to study single myofibril contractility and elasticity is based on a modified atomic force microscope [16], [17]. The later setup did not demonstrate any practical advantages over the original one in terms of system throughput and signal-to-noise ratio. In both cases the principle of the measurement of force in single myofibrils is based on the use of a L-shaped cantilever as a force-sensing element. In the first case the cantilever acts as an obstacle creating a shadow on a photodiode sensor, in the second case the cantilever reflects a laser beam onto a photodiode sensor. In both cases the changes in total proportion of light on the two halves of a split photodiode are used to calculate the deflection of cantilever. As the typical response time of small photodiodes is less than 10 ns, such a force transducer can be used to study very fast dynamic processes. This approach has demonstrated its high efficiency in studies of myofibril contractility [9], [18], [19], [20]. However this method is still not widely used. One of the reasons is that detailed mechanical designs of the apparatus and many techniques and methodological aspects have not been described in sufficient detail. Moreover, to our best knowledge, software for apparatus control and data analysis, which would facilitate further development in this area of research, has not been described.

Here we describe our setup for single myofibril measurements, providing the experimental methodology and describing the software for data acquisition and analysis. The basic system is further enhanced by adding controllability of muscle length, multi-solution changer, real-time analysis of sarcomere length, and software support. The apparatus can be used as a versatile instrument for studying muscle contractility and mechanics. By changing the compliance of cantilever tip it is easy for adapting the system to larger systems such as single cardiac cell studies.

In this paper, we also demonstrate the potential of this by presenting a novel approach to the study of contractility in cardiac myofibrils. As we know, measurements of myofibril contraction are routinely done in an isometric regime. In contrast, natural contraction and relaxation are naturally accompanied with the changes in muscle length. This is particularly important in studies of contractility of heart where the contraction-relaxation cycle is coupled with the heart volume changes. This is an important factor because cardiac preload (stretching in diastole) increases the cardiac response in systole – the Frank-Starling mechanism. We can simulate the cardiac cycle by coupling the timing of changes in Ca^2+^ concentration with the myofibril stretch and release.

## Materials and methods

### Human cardiac tissue samples

Human heart samples were donor hearts samples, for which no suitable recipient was found, from St. Vincent's Hospital, Sydney, Australia. Donor hearts had no history of cardiac disease and normal ECG and ventricular function and were obtained when no suitable transplant recipient was found. Ethical approval was granted by the Research Integrity, Human Research Ethics Committee, University of Sydney (Protocol No 15401) for collection and distribution of the human heart samples and by the NHS National Research Ethics Service, South West London REC3 (10/H0803/147) for study of the samples. Written consent was obtained with PIS approved by the relevant ethical committee. The investigation conformed to the principles outlined in the Declaration of Helsinki.

### Mouse cardiac tissue samples

Experiments and animal handling were done in accordance with the guidelines of the Imperial College London. Mice were killed by cervical dislocation as required by Schedule I of the UK Animals (Scientific Procedures) Act 1986. The left ventricle was dissected and snap frozen before storage in liquid nitrogen. In accordance with the United Kingdom Animal (Scientific Procedures) Act of 1986, this study did not require a Home Office project license because no regulated procedures were carried out.

### Preparation of myofibrils

To prepare myofibrils we used a method described earlier [9], [21]. 1–3 small pieces of muscle samples (total weight of 2–5 mg, but can be as small as 1 mg), frozen in liquid nitrogen, were immersed and quickly mixed-up in a 2 ml eppendorf LoBind tube filled with ice-cold permeabilisation solution. The permeabilisation solution contained (mM): Tris 10 (pH 7.1), NaCl 132, KCl 5, MgCl_2_ 1, EGTA 5, dithiothreitol (DTT) 5, NaN_3_ 10, 2,3-butanedione-monoxime (BDM) 20, and 1% peroxide- and carbonyl-free Triton X-100. BDM in the preparation solution prevented the prepared myofibrils from shortening and increased storage time. The following protease inhibitors (μM) were also added: chymostatin 10, pepstatin 5, leupeptin 10 or 40 (40 mM were added if myofibrils were used for mechanical studies), E-64 (trans-Epoxysuccinyl-l-leucylamido(4-guanidino)butane) 10, and PMSF (phenylmethlysufonyl fluoride) 200. The tube containing the sample was placed on a shaker under slight agitation on ice for 3 h. After permeabilization the solution was removed by pipette and the permeabilised sample was washed by incubation in washing solution (as permeabilisation solution but without Triton X-100 and BDM) for 15 min and then the sample was homogenized for 15 s with a Ultra-Turrax disperser T8 at speed setting 5 (S8N-5G dispersing tool, IKA Werke GmbH & Co., Staufen, Germany) to produce a suspension of myofibrils. The suspension was left on ice for 5 min to let myofibrils to settle down. Then the myofibrils were pelleted by low speed centrifugation. We used a MSE Micro Centaur centrifuge (Sanyo, UK) at low speed for 1–2 min. A supernatant was pipetted out leaving a pellet containing myofibrils. About 300 μL of fresh washing solution was added to the tube and the pellet was re-suspended by pipetting in and out and kept on ice for use in experiments for up to 5 days.

### Instrumentation overview

Our apparatus for single myofibril studies was constructed around an inverted microscope (Eclipse Ti-U, Nikon UK Ltd., Surrey, UK). A CCD camera (Rolera XR, Qimaging, Surrey, Canada) was connected to the left port of the microscope. Two micromanipulators (MP-285, Sutter Instruments, Novato) were mounted on the left and on the right side of the microscope using either custom-made or MT-71-12 (Sutter Instruments) micromanipulator stand. In some experiments a manual micromanipulator (Huxley type) was used instead of the motorized. The complete setup was positioned on an anti-vibration table (63-564, TMC).

### Experimental procedure

A small volume (10–30 μL) of myofibril solution was pipetted onto a coverslip of a custom made temperature-controlled bath chamber, which was positioned on the microscope stage. The bath chamber was made of anodized aluminum with water circulation flow channels for additional temperature control using a thermostatic water bath circulator. The bath chamber had a round hole in the center that was closed by a coverslip. After myofibrils were added, the bath chamber was filled with relaxing solution supplemented with the above mentioned protease inhibitors. Most of the myofibrils slightly adhered to the coverslip. Using two specially prepared fine glass microtools, each mounted onto a micromanipulator, a selected myofibril or small bundle were picked up and positioned horizontally.

The solution in the bath chamber was kept at about the same level during experiments using a peristaltic pump (Instech P720, Harvard Apparatus, USA) in suction mode. The experiments were performed in a temperature-controlled room at 17 °C.

### Experimental solutions

Relaxing (0.01 μM Ca^2+^) and activating (3.16–0.1 μM Ca^2+^) solutions contained (mM): MOPS 10 (pH 7.0), MgATP 5, free Mg^2+^ 1, DTT 5, phosphocreatine 10, and creatine kinase (200 U/mL), bacterial purine nucleoside phosphorylase (200 U/mL), 7-methylguanosine 0.3. The Ca-EGTA:EGTA ratio was set to obtain 10 mM total EGTA and the desired free [Ca^2+^]. Potassium propionate and sodium sulphate were added to adjust the ionic strength of the final solution to 200 mM. Passive stiffness was measured in Ca^2+^-free relaxing solution containing also 30 mM BDM.

### Preparation of microtools

Both glass microtools were prepared from capillary glass tubing (borosilicate glass, 1 mm in diameter) using a pipette puller (PC-10, Narishige) and microforge (MF-900, Narishige). To prepare the L-shaped cantilever force probe, the glass tubing was pulled to obtain a pipette with a tip at least 1.5 mm long and with a nearly constant diameter of 10–12 μm. The extra material was cut off and the open end of the pipette was closed by heating. The pipette was bent by heating to a right angle at 1.2–1.5 mm from the tip, forming a strain sensitive cantilever. This glass tool acted as a force sensor. Another, straight glass microtool also had a tip of about 10 μm but the taper was short. This microtool was much stiffer and its function was to control the position of the other end of the myofibril. This stiff microtool was mounted onto a piezo actuator from the left side and was used to change myofibril length.

To increase the contrast of the projected image on the photodiodes, the region from the tip to the bend was blackened using the microforge. We used the following technique for that: the cantilever part was positioned vertically, at some distance from the heating wire (about 70–90 μm) and then high heat was turned on; the cantilever was quickly progressively blackened during several seconds likely due to the platinum oxide deposition from the heater wire onto the relatively cold glass surface.

### Manipulation of myofibrils with a pair of microtools

Each micromanipulator held a specially prepared microtool, which was used for holding and positioning the myofibrils. The right micro-tool also acted as cantilever force sensor. The left micro-tool was attached to a piezo actuator to change the length of the mounted myofibril.

The technique for myofibril attachment is shown in the Supplementary Video (Video 1). Firstly, the left glass microtool was positioned above a myofibril. A slight touch of one myofibril end by the tip of the left straight microtool was sufficient for firm attachment of the myofibril to the tool. Then the attached myofibril end was lifted up using the micromanipulator whereas the opposite end of the myofibril stayed attached to the coverslip. Holding one end of the myofibril above the cover slip surface, the cantilever tip (right-sided micromanipulator) is brought under the myofibril. A gentle swiping motion of the cantilever tip on the myofibril surface causes the myofibril end to attach to the cantilever tip. The myofibril is then slightly stretched and lifted off the cover slip. The free end of the myofibril wraps around the cantilever tip. Such wrapping increases the area of contact and improves attachment between cantilever tip and myofibril. At the same time, the position of left microtool is continuously adjusted to avoid overstretching of the myofibril. Finally, the position of the two glass microtools is adjusted to ensure that the myofibril is suspended in a horizontal plane.

### Force transducer

The beam of a 5 mW HeNe laser (Lasos, LGK 7628) was directed via the microscope condenser onto a force probe cantilever by means of two adjustable mirrors, The image of the cantilever tip was projected via the microscope optical system (40× objective and 1.5× intermediate magnification) between the two light sensitive areas of double photodiode (Spot-2D, UDT Sensors, Hawthorne, CA). The laser beam illuminated the photodiode cells and generated a current that was proportional to the intensity of the light illuminating the corresponding photodiode cell. The current output of the photodiodes was dependent on the displacement of the cantilever. The current difference between the two photodiodes was proportional to the deflection of the force probe.

The signal inputs from each photodiode cell were differentially amplified, then subtracted and the signal difference was recorded. For this purpose we used a dual operational amplifier (OP270FZ) with a pairs of gain resistors (200 kΩ, RC55Y) as current-to-voltage amplifiers. A differential operational amplifier AD624CDZ was used to find the difference of signals. The circuit was fed with a precision power supply. For power supply decoupling, 6.8 μF tantalum capacitors were soldered between the each power supply terminal and the ground. In addition, 0.1 μF ceramic capacitors were placed close to the power supply terminals of every operational amplifier. The circuit with bi-cell photodiode and signal amplifier was mounted onto a XY translation stage with through-hole (made of NT33-488, Edmund Optics). The XY translation stage was connected to the right side microscope port with a 40 cm length tube. The total resulting magnification of cantilever on photodiodes was 121.4. The translation stages were used to adjust the position of the photodiode so the projection of cantilever was right between the two split photodiode elements. For easy positioning, the shadows of the cantilever tip and photodiode were both projected onto a white surface screen behind.

### Measurement of cantilever force probe compliance

The compliance of each cantilever force probe was measured by comparing it against a calibrated micro-force generator (see below). The micro-force generator pushed the end of the cantilever with different forces and the ratio of tip deflection to force was calculated. The prepared cantilever force probe had the compliance of 2–17 μm/μN when deflected by small forces pulling perpendicularly. The deflection of the cantilever tip is proportional to the force acting on the end of the tip and is detected with the optical system.

### Micro-force generator to measure cantilever compliance

A moving coil meter is used as the force-generating element of a micro-force generator [15]. The torque produced by the coil in the magnetic field is proportional to the current within the range of rotation of the pointer. For our setup we used a conventional ammeter (Sifam, RS-components, 1968418). The ammeter was powered by a regulated DC power supply (Thurlby Thandar, PL320). A resistor (474 MΩ) in series was added to decrease the current. The current flow in the coil depends on the resistor and is proportional to the applied voltage. To calibrate the ammeter response we used it firstly as a balance scale, equilibrating the magnetic force with the gravitational force (known weight). For this purpose, the ammeter was placed in upright position with the pointer aimed upwards. A series of small pieces of thin copper wire of known weights (range 0.1–2 mg) were suspended on the pointer tip and the applied voltage (current) was increased until the pointer returns to its original neutral position on the scale. The force generated by the pointer (in μN) was plotted against the applied voltages. A straight line was fitted to the data and the slope was determined. Such a calibrated ammeter, with known output force per voltage (1.290 ± 0.03 μN/V, *R*^2^ = 0.9999) was used as a micro-force generator.

### Calibration of force transducer

To calibrate the photodiode response, the double photodiode sensor was moved using the micrometer of the vertical translation stage on which it was mounted in steps across the projection of the cantilever force probe. The photodiode output signal was measured to find the voltage–displacement conversion factor (usually ∼0.8 μm/V). The differential response of photodiodes to cantilever movement was recorded before each experiment. An alternative method for calibrating photodiode response is to directly move the cantilever force probe by synchronous movement of the two micromanipulators.

### Ultrafast two solutions change system

Myofibrils were activated and relaxed by step changes in Ca^2+^ concentration achieved by moving a double-barrelled micropipette across the mounted myofibril. The pipette was pulled from Θ glass capillary (TGC200-15, Clark Electromedical Instruments), incised and broken along the line using a diamond glass cutter, and, if required, polished by hand with a fine polishing stone. The final outer diameter of the prepared Θ pipette was 300–500 μm. The pipette was clamped on a holder mounted onto the spline shaft of a stepper motor (Z26544-05, Haydon Kerk) with a linear travel of 0.102 mm per step and with the velocity of almost 5 ms per step. The system was mounted onto a 3-axis positioning stage (combined from M-433 translation stages, Newport). Tubing was inserted into each septum and glued. The other ends of the tubing were connected to syringes filled with activating and relaxing solutions. Solution flow was driven by gravity and the flow rate was controlled by the height between the reservoirs and the experimental chamber (with the flow rate about 0.3 ml/s). The micropipette was positioned perpendicular to the long axis of the myofibril. Both channels of the double pipette were continuously perfused with relaxing and activating solution during experiments. At the initial position of the pipette, a flow of relaxing solution running from one pipette barrel was aimed directly onto the myofibril. To quickly change solutions, the motor moved the pipette horizontally (by ∼0.2 mm) switching the solution streams directed toward the mounted myofibril. The time for solution switch to complete was less than 10 ms.

### Multiple solution change system

As one barrel of Θ pipette was constantly perfused with relaxing solution, the second barrel could be perfused with any one of 8 solutions. An 8-channel valve (HVXM 8-5, Hamilton, VWR, Lutterworth, UK) regulated which activation solution was supplied. The valve has 8 inlets but only one outlet. A stepper motor (21NRLH-LNN-NS-00, National Instruments, UK), controlled via a stepper drive (P70530, National Instruments, UK), changed the position of the valve plug.

### Myofibril length actuator

The myofibril length was altered via the left glass microtool mounted onto a piezo-actuator using a B-8B ball joint adapter (without attachment bar) and UPN-C pipette holder (Narishige). The piezo actuator (P-212.4S, Physik Instrumente; controlled by E-471.20 amplifier) was equipped with a servo-control module E-509.S1 for closed-loop operations. The function of such length actuators was automatic myofibril length regulation in fast and low speed operations such as fast and slow release-restretch, sinusoidal perturbations and cardiac cycle simulations.

The protocol for release-restretch was the following. When a myofibril was activated with high [Ca^2+^] and the force reached a plateau, a fast piezo-electric expansion (20% of the myofibril length in 2 ms) brings the myofibril to the slack state. After a short hold period of 20 ms, the piezo-electric element contracted (in 2 ms) bringing the myofibril back to its original length.

### Data acquisition and apparatus control

Apparatus control and data recording were done using a data acquisition device (NI USB-6251, National Instruments, Newbury, UK) and custom-written software in the LabVIEW 2015 environment (National Instruments, Newbury, UK). The following signals were acquired, and then visualized and recorded: the signal from the force transducer; the actual piezo actuator position; each step command sent to the stepper motor controller of the fast solution change system; and each change in the valve position of the multi-solution change system, where the number of spikes/peaks encoded the valve position. The sampling rate was 1 kHz. The peak-to-peak background noise in the signal from force transducer was 50–300 mV at low frequencies (0.2–10 Hz) and 5–30 mV at higher frequencies

### Sarcomere length measurement

Sarcomere length was monitored in real time using the Fast Fourier Transform of the myofibril image. A region of interests was drawn around the mounted myofibril and its image profile was extracted. The power spectrum of the profile was calculated. The spatial frequencies outside those that were related to the experimental sarcomere length (1–4 μm) were cut-off. The myofibril length was adjusted manually by a micromanipulator to set the mean sarcomere length to the desired value.

### Data analysis

In the data analysis of contraction/relaxation experiments, previously saved experimental data was loaded. Based on the acquired metadata, the program automatically identified the following parameters: Ca^2+^ transients; activating [Ca^2+^] based on the valve position characterizing the concentration of Ca^2+^ in activating solution. All contraction-relaxation cycles were numbered and their activation and relaxation phases were automatically and accurately separated. After calibration parameters were applied, the trace segments were fitted with single exponential or linear fit with instant visual feedback. The edge between the slow relaxation phase and the fast relaxation phase and the segment of the slow phase to be fitted with linear was selected manually using cursors. The maximal tension was calculated from the difference between the baseline and the exponential plateau.

The rate constants for the exponential force development upon activation (*k*_ACT_) and fast phase of relaxation (*k*_REL_) were evaluated by curve-fitting using Levenberg–Marquardt nonlinear least square algorithm in LabVIEW.

$$y=y_{\max}+(y_{0}-y_{\text{plateau}})\exp(-kt)$$

The slow phase can be fit with a linear fit and characterized by the rate constant *k*_LIN_ calculated from the slope of the linear fit by normalizing it to the maximum tension.

$$k_{\text{LIN}}=\frac{-\text{slope}}{y_{\max}}$$

The maximum tension response to different [Ca^2+^] was fit with the Hill equation and [Ca^2+^] for the development of half maximal force response (EC50) and nH can be estimated from this fitting.

$$F=F_{\max}\times \frac{{\left[{\text{Ca}}^{2+}\right]}^{\text{nH}}}{\text{EC}50+{\left[{\text{Ca}}^{2+}\right]}^{\text{nH}}}$$

Data were fitted with the Hill equation in GraphPad Prism 6 (GraphPad Software, San Diego, CA). Statistics analysis (unpaired *t*-test) and graphs were also prepared with GraphPad Prism.

## Results and discussion

### The experimental setup

#### Apparatus design

The measurement principle of the apparatus has already been described earlier and our apparatus is a development of a previous instrument [22]. We show here important improvements in the control of muscle length, of experimental solution changing, in real-time analysis of sarcomere length, and in control software. The main components of the mechanical and optical setup are shown in Fig. 1a–f. The apparatus consists of an inverted microscope, a camera to capture images and two micromanipulators for manipulating and positioning myofibrils by means of two microtools. One of the microtools acts also as a force-sensing cantilever. A laser beam is directed onto the cantilever tip. Under laser illumination, the image of the cantilever is projected through the optical path of the microscope onto a photodiode position detector. The response of the photodiode based force transducer is linearly dependent on the cantilever deflection produced by the myofibrillar force applied to it.

The myofibril state is switched from contraction to relaxation and back by rapidly moving the solution flow bathing the myofibril from one of two solution streams running from the double-barreled Θ pipette to the other. A motor controls the position of an 8-channel valve that regulates which one of eight activating solutions is being delivered. Tubings connect syringes with different activating solutions to the valve inlets. The valve output is fed to one side of the double-barreled Θ pipette. The other side of the pipette is fed with relaxing solution. Solution flow is driven by gravity from the elevated syringes. The motorized valve system to supply multiple solutions is more reliable and with less solution mixing alternative to the systems based on solenoid valves with a multi-barrelled manifold.

#### Apparatus control and acquisition software

The performance of the apparatus critically depends on the ability to control various actuators at specified times with millisecond accuracy. The LabVIEW programming development environment was used for this purpose. Fig. 2 shows the schematics of apparatus control principle and data acquisition via a data acquisition (DAQ) device.

The software allows the automatic measurement of myofibril length and of the mean sarcomere length in real-time; it automates the solution change system (valve selection and position of the Θ pipette); it generates an arbitrary waveform to control the position of the length actuator and controls the timing of the fast solution change system.

The camera view is used to set up the apparatus and to mount the myofibrils. The sarcomere length is calculated in real time from the Fast Fourier Transform of the myofibril image. Supplementary Fig. S1 shows the graphical interface used for sarcomere length analysis. The graphical interface of the control software is shown in Fig. 3. Using the panels for construction of the length change and solution-change protocols, it is possible to apply any combination of length and solution changes and to generate sequences of control commands to automate tasks. The displacement of the length actuator can be easily scaled to adapt to various lengths of myofibril samples. Experiments are viewed in real time and data are saved for analysis. The signals from the force transducer, the fast solution switch system, the position sensor of the piezo actuator and the state of the multiple solution control system are recorded for use in data analysis.

#### Data analysis software

Despite improvements in performance of spreadsheet and commercial statistical software in recent years, they are usually not effective for processing large data sets. Data analysis software was written in LabVIEW to visualize the acquired data, to process it, to fit model curves to key components, to visualize the results and to organize them for further analysis. The data analysis software (Fig. 4) is loaded with saved experimental data. The tension traces are automatically identified, numbered and activation and relaxation events are automatically and accurately selected. The valve configuration and therefore which activating solution is used for each contractile event is automatically determined. The transition between the slow relaxation phase and the fast relaxation phase, and the segment of the slow phase which is to be fitted with a straight line is selected manually using cursors. After calibration parameters are applied, the trace segment is fitted with a single exponential. The fitting intervals can be easily adjusted with the cursors with constant visual feedback. The best-fit parameters are saved and transferred to another statistical program for further analysis.

## Experimental evaluation

### Fast two solution switching system

Rapid activation and relaxation in myofibrils is achieved by switching the stream of experimental solution directed at the mounted myofibril. The solution streams were applied via the two adjacent barrels of a Θ pipette, whose position was controlled by a stepper motor. Single myofibrils as well as small bundles, due their small diameter (<3 μm), equilibrate with the surrounding solution within 1 ms, without being restricted by significant diffusion time lag, so that the observed rates of force development (*k*_ACT_) and of relaxation (*t*_LIN_, *k*_LIN_, *k*_REL_) following quick increase and decrease in Ca^2+^ level, respectively, correspond to the kinetics of the protein/protein and protein/ligand interactions (Fig. 5).

*k*_ACT_ represents the cross-bridge turnover rate which is determined by the dynamic equilibrium between the apparent rates with which cross bridges enter and leave the force generating states. *k*_ACT_ can also affected on by the rate at which thin filaments are switched on by Ca^2+^. Isometric tension is dependent on the number of attached myosins. Relaxation is initiated by Ca^2+^ dissociating from troponin C coupled to the release of the C-terminus of troponin I from troponin C and its attachment to actin, where it blocks cross-bridge binding cooperatively. The lag in the force trace has been ascribed to the time taken for the occupancy of cycling cross-bridges to drop below the threshold for cooperative activation of the thin filament, and the subsequent rapid-relaxation phase corresponds to the detachment of the remaining cross-bridges, where reattachment is prevented in Ca^2+^-free solution. These experimental parameters have been fully described by Stehle et al. [23].

### Myofibril length changes

An important feature of the experimental apparatus presented here is the ability to change myofibril length under computer control. A length transducer, composed of a microtool mounted onto a piezo actuator is used to change the myofibril length. This length actuator makes it possible to use a different approach to the measurement of the rate of tension development. The application of a quick decrease in myofibril length (a quick release) applied during active contraction and immediately followed by a restretch pulls off all actin-attached myosin heads. Immediately after the restretch, the forcibly detached myosin heads reattach and force starts rising again [24] (Fig. 6a and b). The use of myofibrils, compared to the very much thicker muscle fibers (20–150 μm) avoids delays in the rate of force development caused by diffusion of calcium into the fiber lattice which might cause a lag in force development and affect the measurement of *k*_ACT_. In the absence of a diffusion induced lag in Ca^2+^-activation, *k*_TR_ is equal to *k*_ACT_ (*P* = 0.945, Fig. 6c). Comparison of *k*_ACT_ with *k*_TR_ shows that Ca^2+^-switching is not rate-limiting factor for single myofibrils. The time-course of force development observed in myofibrils is therefore simpler than that observed in permeabilised muscle fibers [24].

Control of muscle and sarcomere length also enables us to study length-dependence of measured parameters as well as viscous and elastic properties of myofibrils. For instance, to measure passive stiffness, myofibrils in the relaxed state (in the absence of calcium) were stretched via the microtool attached to the length actuator. The interval between stretch steps was 20 s. The initial sarcomere length was chosen such that the mounted myofibril was not slack. Each myofibril stretch caused an increase in strain, followed by relaxation when the stretching was paused (Fig. 7a). The stretch and the following release shows hysteresis (Fig. 7b) which is explained with the fact that the rate of domain unfolding in the multi-domain protein titin, is slower than the refolding [25]. This explanation can be tested by waiting a long time between releases on the return from the stretch.

### Multi solution change system

This system enables us to determine dose-response relationships. For instance, the tension response of myofibrils to [Ca^2+^] is cooperative and well described by the Hill equation. The relationship between contractility and Ca^2+^ concentration is examined by applying a range of activating solutions of varying [Ca^2+^], and measuring the ensuing maximal isometric force (Fig. 8). Apart from the maximal isometric force, the rates of activation and relaxation are measured over a range of Ca^2+^ concentrations, providing more information about myofibril kinetics as we have demonstrated [9]. Furthermore, Ca^2+^ curves can be obtained at different initial sarcomere lengths to enable us to measure length-dependent activation in single myofibrils [9].

### Cardiac cycle simulation

Combining precisely sequenced length and solution changes allows us to simulate complex contraction patterns as observed during the in vivo heart cycle. The principal mechanical characteristic of cardiac pumping is that contraction-relaxation cycles are coupled with heart volume changes. According to this, the cardiac cycle of the left ventricle can be divided into 4 phases: isovolumic contraction, ejection, isovolumic relaxation and filling. Cardiac contraction is not isometric. During diastole the cardiac wall stretch, whereas during systole the muscles contract. The changes in the left ventricle heart volume are coupled with the changes in myofibril length. Thus, based on the heart volume changes, the 4 phases of the typical human cardiac cycle are (for a heart rate of 70 beats/min): isovolumic contraction (70 ms) and ejection (281 ms), isovolumic relaxation (50 ms), and ventricular filling (461 ms) [26], [27]. Since, at the experimental temperature of 17 °C, the rate of biological processes in muscle is lower, and considering the temperature coefficient *Q*_10_ ≈ 2.6 [28], the time for contraction and filling was increased. The range of sarcomere lengths in the left ventricle during a cardiac cycle varies between 1.8 and 2.3 in mammalian heart [29].

Using the computer-controlled piezo actuator we drove cyclical changes in myofibril length, similar to the changes in myofibril length that occur during a cardiac cycle. The changes in myofibril length were synchronized with the changes in concentration of Ca^2+^ with a lag between activating Ca^2+^ addition and shortening to allow for isometric (isovolumic) contraction (Fig. 9a and b). The area of the loop (Fig. 9c) represents the amount of work done in each cycle and the reduction in tension at the end of the ejection phase with shorter sarcomere length gives a homologue of end systolic pressure volume relationship that indicates the length dependence of contractility.

## Conclusions

The single myofibril force assay system measures the physiological and mechanical parameters in myofibrils such as maximal force, Ca^2+^-sensitivity of tension, rates of contraction and relaxation and myofibril elasticity. By combining programmed length changes and solution changes the apparatus is capable of making many more complex studies such as length-dependent modulation of Ca^2+^-sensitivity or to mimic the cyclic changes in Ca^2+^ concentration and myofibril length to simulate the cardiac cycle.

## Figures and Tables

**Figure:**
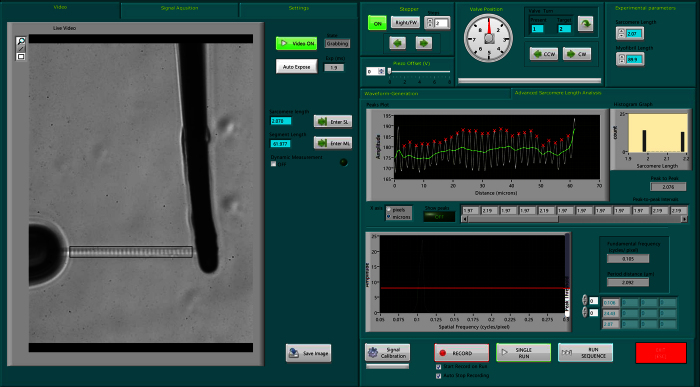


**Fig. 1:**
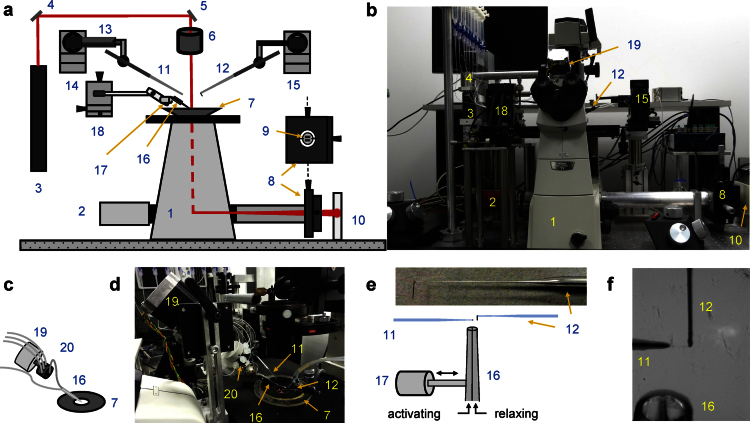
Experimental setup. (a, b) Schematic overview (a) and photograph (b) show the key elements of the apparatus. (c, d) Schematics (c) and photograph (d) of multi-solution change system. (e) Schematics of cantilever and ultrafast solution change system. The left-sided microtool is relatively stiff and is mounted onto a piezo actuator for fast myofibril length change. The right-sided glass microtools is a high compliance L-shaped cantilever that acts as a force sensor. The inset shows a photograph showing the shape of the cantilever tip. (f) The photograph shows two microtools holding a small myofibril bundle. The relaxing and activating solutions are applied via the adjacent barrels of a double-barreled (Θ) micropipette. The numbers on the figure indicate: 1 – inverted microscope; 2 – camera; 3 – laser; 4 and 5 – mirrors; 6 – microscope condenser; 7 – bath mounted onto a microscope stage; 8 – XY positioning stage; 9 – segmented photodiode; 10 – projection plane for visual control of cantilever positioning; 11 – left microtool; 12 – right microtool (cantilever force sensor); 13 – piezo actuator; 14 and 15 – left and right micromanipulators for the positioning of left and right microtool, respectively; 16 – double-barreled Θ pipette; 17 – stepper motor controlling the position of the double-barreled Θ pipette; 18 – XYZ positioning stage for the ultrafast two solutions switching system; 19 – stepper motor controlling the valve opening of the eight-channel valve; 20 – eight-channel valve.

**Fig. 2:**
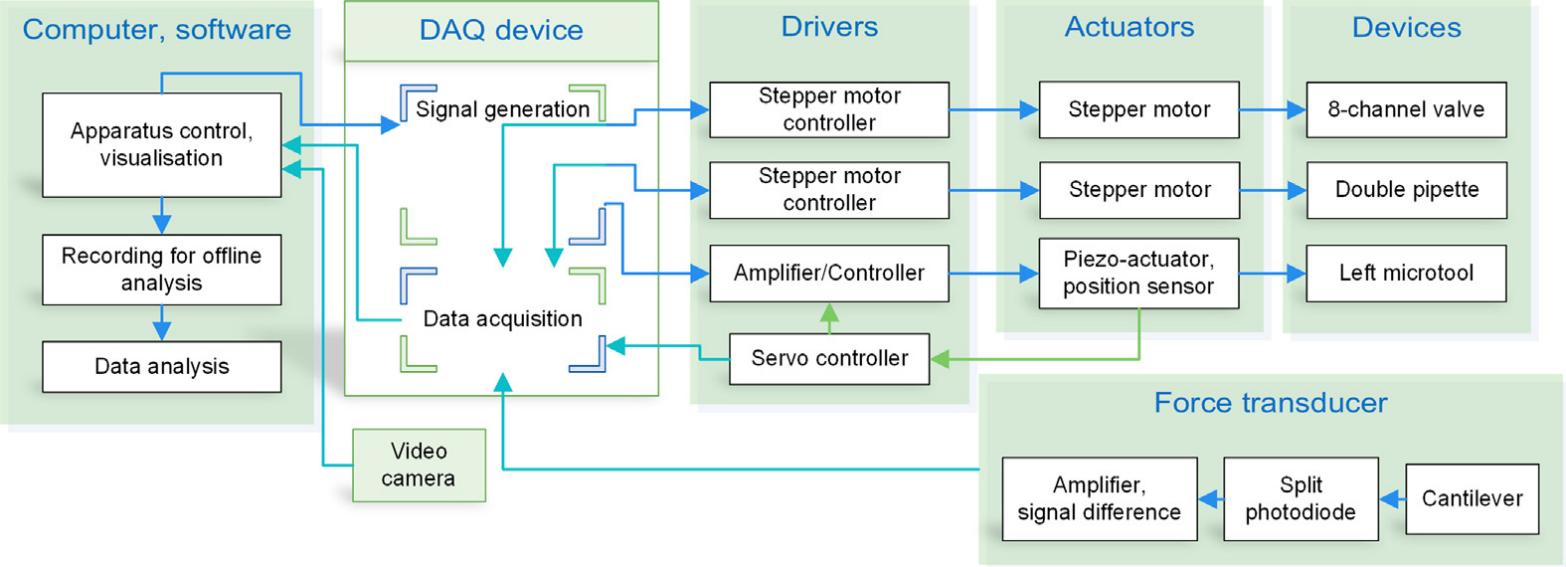
Block diagram of apparatus control, data acquisition and analysis. Arrows show the signal/information flow between components.

**Fig. 3:**
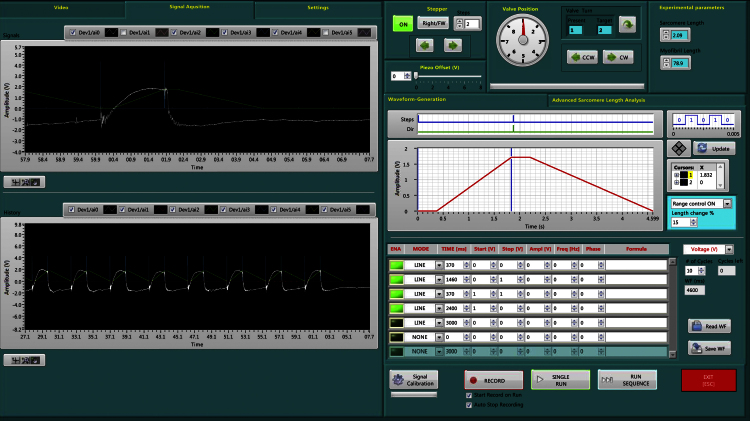
Software interface used for apparatus control. The graphs on the left are used to visualize acquired data from the photodiode position sensor, the solution change system and length actuator. The graphs and panels on the right are used for the construction of length change and solution-change protocols. It is possible to generate a sequence of control commands and apply almost any combination of length and solution changes. The control protocol can be saved in a file to recall later. The experimental record shown is that from a single human cardiac myofibril subjected to length and solution changes that simulate the heart cycle.

**Fig. 4:**
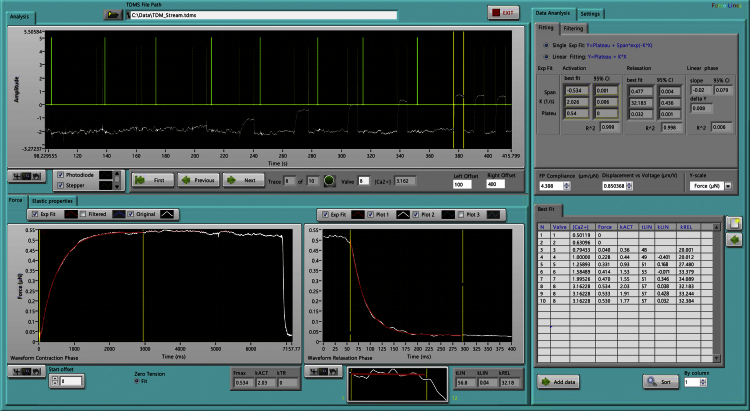
The user interface of the data analysis program. This trace shows a Ca^2+^ curve experiment. The tension traces are automatically identified, numbered and activation and relaxation events are automatically selected.

**Fig. 5:**
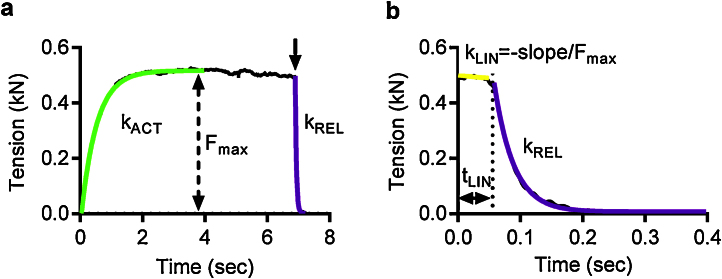
The time course of contraction and relaxation. (a) An example of a contraction-relaxation trace in a single mouse cardiac myofibril. Tension rises without a notable lag when [Ca^2+^] is increased. Tension development is fitted with a single exponential and the rate of contraction (*k*_ACT_) and the maximum tension are calculated as the best-fit parameters. (b) Relaxation following a decrease in [Ca^2+^] is biphasic; the initial slow and nearly linear relaxation phase is followed by a fast phase which is well fit by a single exponential function. To characterize the overall myofibril relaxation, the duration of the slow relaxation phase (*t*_LIN_), the normalized slope (slope divided by *F*_max_ value) of the best-fit line and the rate of the fast relaxation phase (*k*_REL_) are calculated.

**Fig. 6:**
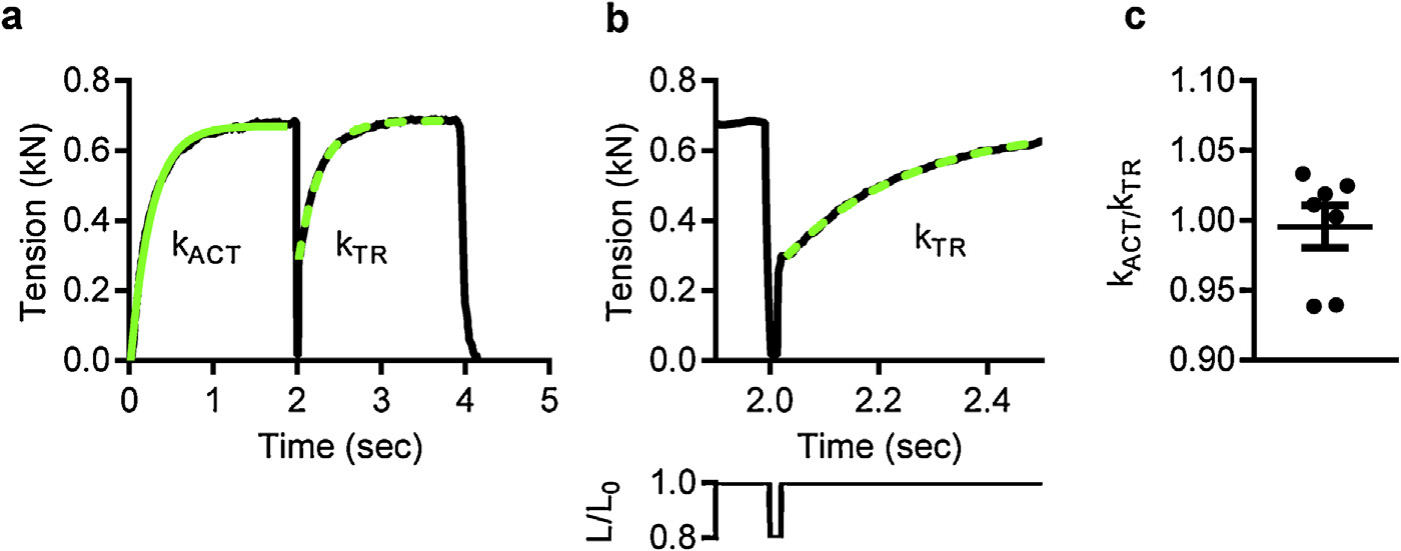
Release-restretch protocol applied to mouse cardiac myofibrils. (a) An example of the application of the release-restretch procedure to a small myofibril bundle. The restretch is applied 20 ms after the end of the 20% length release. (b) The release-restretch is shown on an expended time-scale with the length change protocol shown below. *L*/*L*_0_ is the ratio of the ongoing myofibril length to the initial length. (c) *k*_TR_ is not significantly different from *k*_ACT_. *k*_ACT_/*k*_TR_ values (closed circles) were calculated for 7 different myofibrils. The mean and standard error is shown.

**Fig. 7:**
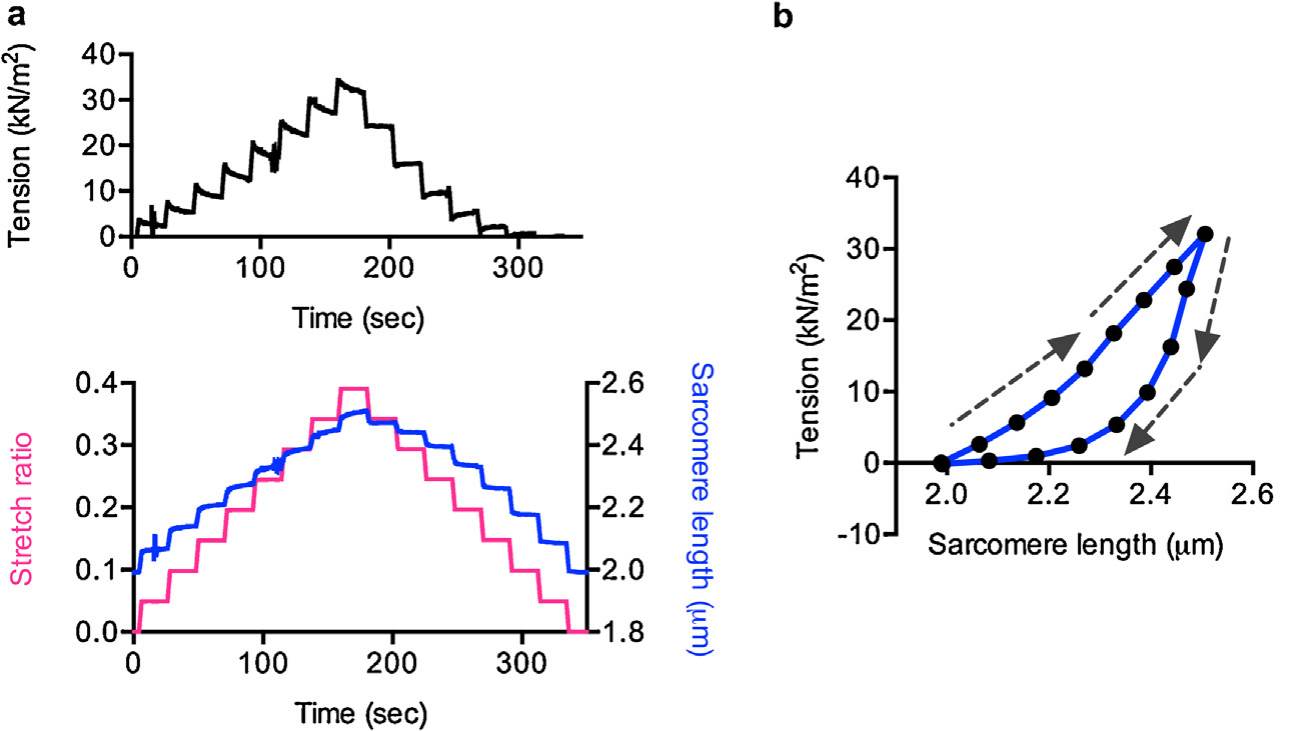
Mechanical properties of human cardiac myofibrils. (a) Passive tension changes (upper plot) in response to incremental stretch and release (lower plot). (b) The relationship between sarcomere length and passive tension. The arrows show the direction of the sarcomere length change.

**Fig. 8:**
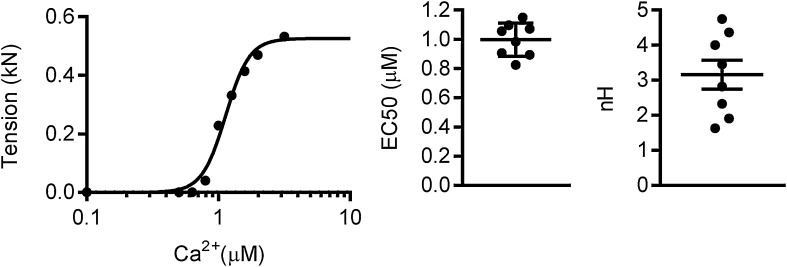
The Ca^2+^-sensitivity of tension in mouse myofibrils. Representative *F*_max_ – [Ca^2+^] curve obtained from a small myofibril bundle of 2–8 myofibrils. The Hill equation was fitted to the [Ca^2+^]-tension response and the best-fit parameters – EC_50_, namely the [Ca^2+^] for the development of half maximal force response, and Hill slope (nH), the measure of cooperativity, were calculated for each myofibril (*n* = 8). Horizontal bars indicate the means with standard errors.

**Fig. 9:**
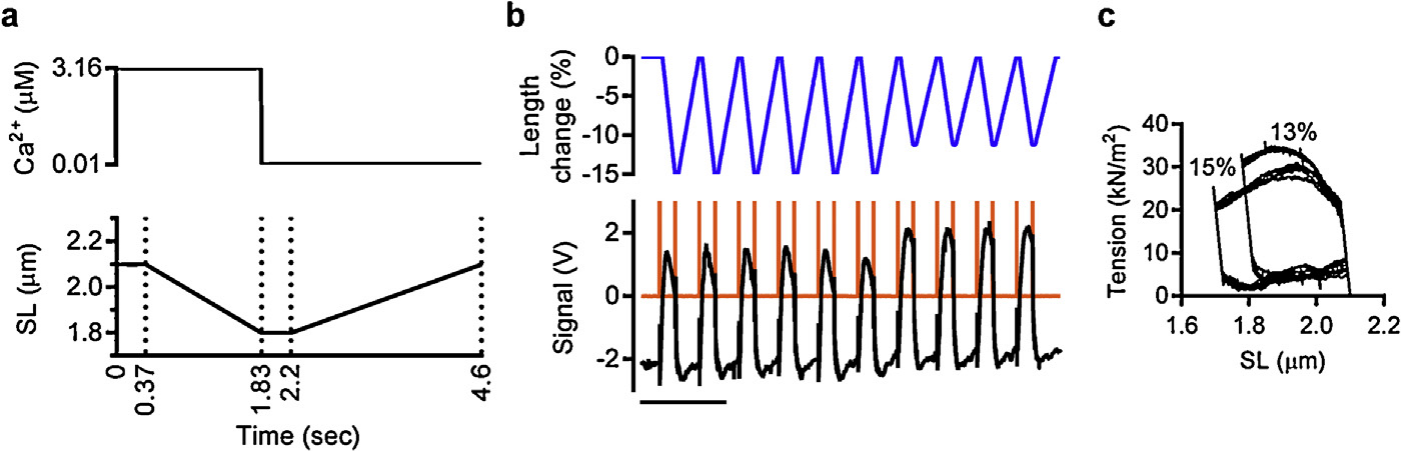
Simulation of the heart cycle in human cardiac myofibrils. (a) A model for simulating heart cycle dependent changes in sarcomere length. The vertical dotted lines separate 4 cardiac phase: isometric contraction, ejection, isometric relaxation and filling. (b) The imposed myofibril length change is shown by the blue line, myofibril tension in black. 3–4 V spikes in orange show the solution change events. Scale bar is 10 s. (c) The developed tension is plotted against sarcomere length for several cycles. The imposed length change is 13% in the initial 6 cycles and 15% of the initial sarcomere muscle length in the last 4 cycles.
