# Supplementary figures and images for: Instrumentation to study myofibril mechanics from static to artificial simulations of cardiac cycle
Source: MethodsX. 2016 Mar 2;3:156–70. doi: 10.1016/j.mex.2016.02.006 (PMC4796715; doi:10.1016/j.mex.2016.02.006)

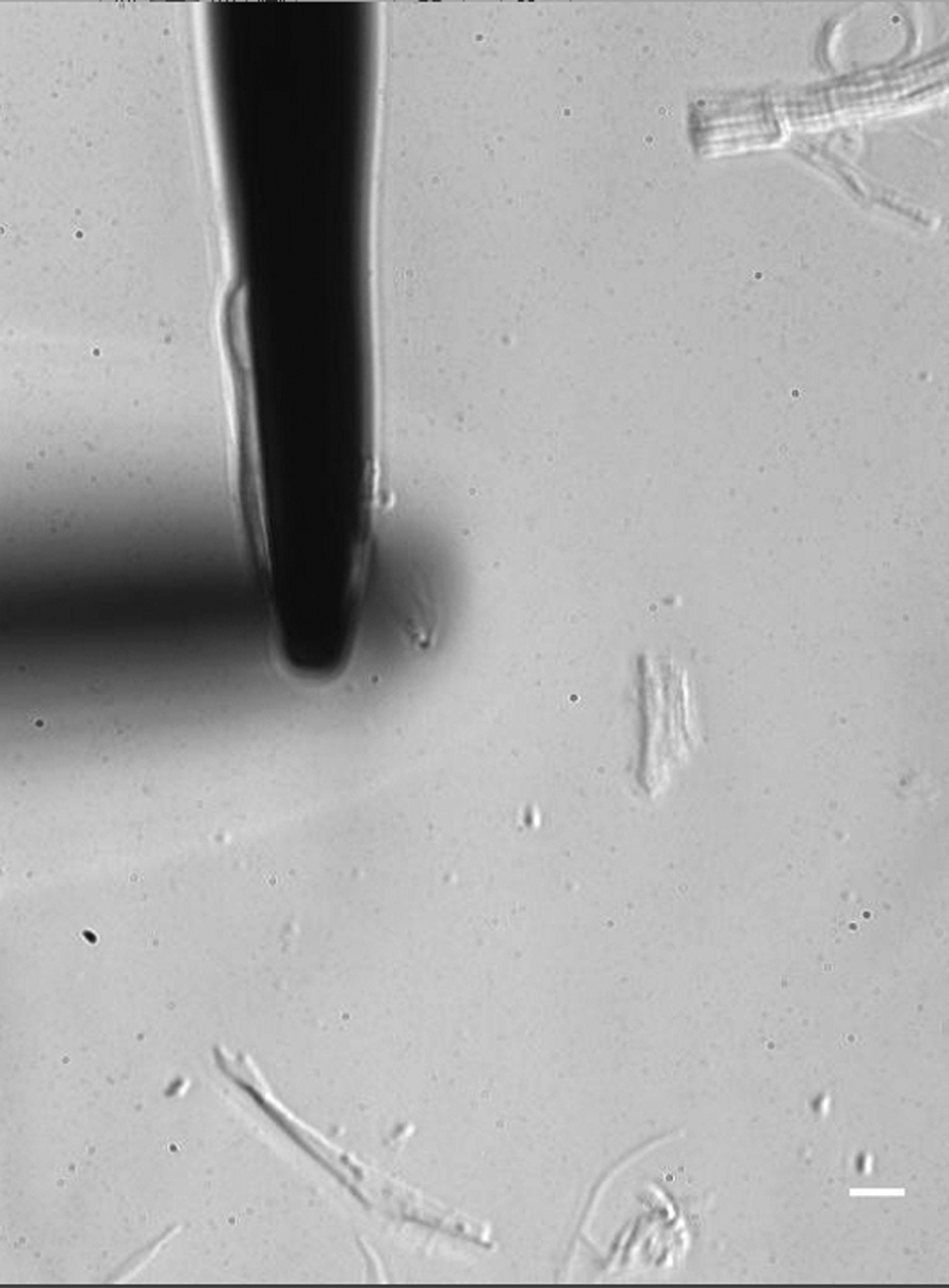

Supplement: Supplementary file 1 [file mmc2.jpg]
